# Research and Remedies

**Published:** 1912-10-05

**Authors:** 


### RESEARCH AND REMEDIES.

### The Latest Method of Ether Anæsthesia.

Inventions for administering ether arrive so rapidly nowadays that the heading of this paragraph may be erroneous before it can appear in print. The intratracheal insufflation of a warmed, moistened, and ether-charged air current, supplied by a motor and entirely independent of the working of the natural respiratory pump, is at least novel for the moment. It has been tested in this country by R. E. Kelly, of Liverpool, whose recent paper in the *British Medical Journal* summarises the work of several American investigators who have devoted attention to the method. In principle, the idea is to supply through an intratracheal cannula a stream of air containing ether, and to deliver it at about the bifurcation of the trachea, whence it reaches the alveoli by diffusion; originally conceived for the requirements of thoracic surgery, the plan has now been tried also for throat and jaw work. The machinery necessitated is complicated and expensive, but probably less so than the Sauerbruch cabinet for thoracic surgery, which it renders unnecessary. We commented a short time ago on the reluctance of British surgeons to adopt this cabinet; but, if this method of anæsthesia turns out to fulfil the hopes of its inventors, our hospitals will be able to save their money. For a detailed description of the plant required, the original paper should be consulted; but it may be noted that in experimental work on animals in the United States it has proved possible to remove one or more lobes of a lung, to divide and suture the œsophagus, and to divide and suture the arch of the aorta!

### A New Hypnotic.

Of late years the deluge of new hypnotics, each superior to all predecessors, has largely subsided; and it is becoming quite an uncommon event for a serious rival to the old-established favourites to lift its head. Recently, however, a new drug of this class has been put on the market under the trade name of codeonal. It is stated to be a mixture of codeine diethyl-barbiturate and sodium diethyl-barbiturate in the proportions of 11.76 and 88.24 per cent. respectively. The mixture is manufactured in tablets of about 2½ grains each, of which one or at the most two is suggested as the normal dose. Several advantages are claimed for the combination, especially freedom from after-effects. The substance is especially recommended as a hypnotic and sedative in cases where sleep is interfered with by pain, cough, etc. It is natural that the manufacturers or vendors of a new hypnotic should take an optimistic view of its merits; and it is to be hoped that independent verification of their claims will be forthcoming from some unprejudiced authority.

### Anæsthesia by Paraldehyde.

As a hypnotic paraldehyde has many virtues which have long been recognised by the medical profession; but its nauseous taste has proved a drawback to its popularity which has prevented it from competing on equal terms with trional, veronal, and similar drugs. Hitherto it has not been used except as a simple hypnotic; but Messrs. Noel and Soutar, of the London Hospital, have been experimenting with it by intravenous injection as an anæsthetic. They find it extremely useful for short operations, especially in alcoholic or other difficult subjects, because its action is so extremely rapid. Unconsciousness is complete in forty seconds, and deep anæsthesia with loss of reflexes in fifty seconds more. Reflex movements may, however, even then occur, which seems to limit the utility of the method to that class of cases for which nitrous oxide and ethyl chloride are now so generally employed. The method employed is to mix 5 to 15 c.c. of paraldehyde with an equal amount of ether, and then to dissolve the mixture in 150 c.c. of a sterile 1 per cent. saline solution, free from dead bacteria. The solution is run into one of the veins of the fore arm through an apparatus similar to that commonly employed for salvarsan at a rate of 30 to 60 c.c. per minute. About 5 to 10 c.c. of paraldehyde are required to produce anæsthesia deep enough for the removal of teeth, the suturing of scalp wounds, and similar casualty-room operations. Consciousness is sometimes fully regained in twenty minutes or so, but sometimes the patient passes into a natural sleep which may last for some hours.

### Three Digitalis Preparations.

In the *Biochemical Journal* Dr. Douglas Cow sums up the results of his experiments with three proprietary digitalis preparations now on the market, which are extolled by their respective manufacturers as superior in various respects to ordinary tincture of digitalis. *Digitalone*, he finds, is in every respect inferior to tincture of digitalis as a cardiac stimulant; indeed, when it is administered by the alimentary canal there is no evidence that it exerts any physiological action on the circulatory system. *Digalen* is less toxic than tincture of digitalis when perfused through the heart; but if administered by the mouth this difference is not evident. It increases the amplitude of the heart-beat to the same extent as digitalis, but is inferior in that it does not slow the beat so much. The mean tonus of the heart is raised by digalen, thus involving a large expenditure of energy which may be useless, and though it can conceivably be advantageous, as in acute dilatation, in ordinary cases is likely to be harmful. When given subcutaneously it is more irritating than any other preparation of digitalis, and it is by no means cheap. *Digipuratum* is more toxic than tincture of digitalis when perfused through the heart, and it markedly diminishes the rate of coronary flow. It slows the heart rather more than the tincture does; in the matter of increase of amplitude there is little to choose between them. It acts quite well when given by the alimentary canal, but is quite as irritating as digitalis when given subcutaneously. Tincture of digitalis it appears, concludes Dr. Cow, is not to fear for its superior.

